# Co-design of a patient experience survey for arthritis central intake: an example of meaningful patient engagement in healthcare design

**DOI:** 10.1186/s12913-019-4196-9

**Published:** 2019-06-04

**Authors:** Eloise C. J. Carr, Jatin N. Patel, Mia M. Ortiz, Jean L. Miller, Sylvia R. Teare, Claire E. H. Barber, Deborah A. Marshall

**Affiliations:** 1 Faculty of Nursing, University of Calgary, PF2237, 2500 University Drive NW, Calgary, Alberta T2N 1N4 Canada; 2 Pan-SCN Manager, Strategic Clinical Networks™, Alberta Health Services, 10030 – 107 Street NW, Edmonton, Alberta T5J 3E4 Canada; 3 O’Brien Institute for Public Health, University of Calgary, 3280, Hospital Dr. NW, Calgary, Alberta T2N 4Z6 Canada; 4 Arthritis Research Center, University of Calgary, HRIC 3AA20, 3280, Hospital Dr. NW, Calgary, Alberta T2N 4Z6 Canada; 5 Cumming School of Medicine, University of Calgary, Health Research Innovation Centre (HRIC) – 3C56, 3280 Hospital Drive NW, Calgary, Alberta T2N 4Z6 Canada

**Keywords:** Arthritis, Patient experience, Patient engagement, Co-design

## Abstract

**Background:**

To describe the process of patient engagement to co-design a patient experience survey for people with arthritis referred to central intake.

**Methods:**

We used a participatory design to engage with patients to co-design a patient experience survey that comprised three connected phases: 1) Identifying the needs of patients with arthritis, 2) Developing a set of key performance indicators, and 3) Determining the survey items for the patient experience survey.

**Results:**

Patient recommendations for high quality healthcare care means support to manage arthritis, to live a meaningful life by providing the right knowledge, professional support, and professional relationship. The concept of integrated care was a core requirement from the patients’ perspective for the delivery of high quality arthritis care. Patients experience with care was ranked in the top 10 of 28 Key Performance Indicators for the evaluation of central intake, with 95% of stakeholders rating it as 9/10 for importance. A stakeholder team, including Patient and Community Engagement Researchers (PaCER), mapped and rated 41 survey items from four validated surveys. The final patient experience survey had 23 items.

**Conclusion:**

The process of patient engagement to co-design a patient experience survey, for people with arthritis, identified aspects of care that had not been previously recognized. The linear organization of frameworks used to report patient engagement in research does not always capture the complexity of reality. Additional resources of cost, time and expertise for patient engagement in co-design activity are recognized and should be included, where possible, to ensure high quality data is captured.

**Electronic supplementary material:**

The online version of this article (10.1186/s12913-019-4196-9) contains supplementary material, which is available to authorized users.

## Background

Globally, osteoarthritis (OA) causes 12.8 million years lost to disability (YLDs) and is ranked 13th, in the top causes of top causes of global YLDs in 2013, ranked higher than cancer (6.7 million YLDs) and hypertensive heart disease (11.9 million YLD) [1]. Osteoarthritis is the most common form of arthritis, and there is no cure. OA affects approximately 12.5% of people in Canada and rheumatoid arthritis (RA) 1%, causing pain, disability, and a significant human burden [2]. Unlike OA, early detection and referral to specialist services for people with RA can significantly affect outcomes [3] but established wait time benchmarks are rarely achieved [4]. A survey of 4565 Canadians aged 20 or older, found that the average time between onset of symptoms and OA diagnosis was 7.7 years [5]. During this period, patients can experience pain, depression, anxiety and increasing disability [6]. Understanding the patients experience as they wait for specialist services can help shape the delivery of health care to provide care that is commensurate their needs. For example, in a qualitative study, using a patient-to-patient method where patient researchers with OA engaged others with OA to co-develop a model for the referral to specialist services, the most salient, overarching theme was ‘supporting us managing a meaningful life with arthritis’ [7].

When patients are engaged as researchers they bring their health experiences and as partners in healthcare system design can improve the quality of care, and enable healthcare providers and administrators to meet the needs of a growing number of patients living with chronic health conditions [8, 9]. In the UK, patient and public involvement in health-care planning, service development, healthcare policy and research have gained growing importance over the past two decades [10]. In Canada, several initiatives have embraced patient-centered care approaches but change has been slow [11]. However, in Alberta, a number of initiatives have firmly position patients in the decision-making process when shaping healthcare redesign [12, 13].

One example of a project that has consistently engaged patients in decision-making relates to the provision of arthritis care. Funded by Alberta Innovates (AI), Alberta Health Services (AHS), the Arthritis Society, and the Canadian Institute for Health Research, “Optimizing Centralized Intake to Improve Arthritis Care”, has a specific objective to identify the optimal referral management and triaging strategy across varying central intake processes in Alberta. Central intake (CI) systems are single point-of-entry for referrals, usually from primary care to a health care service, with the aim to streamline services, reduce bottlenecks and reduce wait times for specialist assessment [14]. Models such as these have the potential to improve timeliness and patient-centeredness of elective surgical procedures [15] and have been proposed as one of the approaches to tackle waiting time for patients with suspected RA [16].

**Fig. 1:**
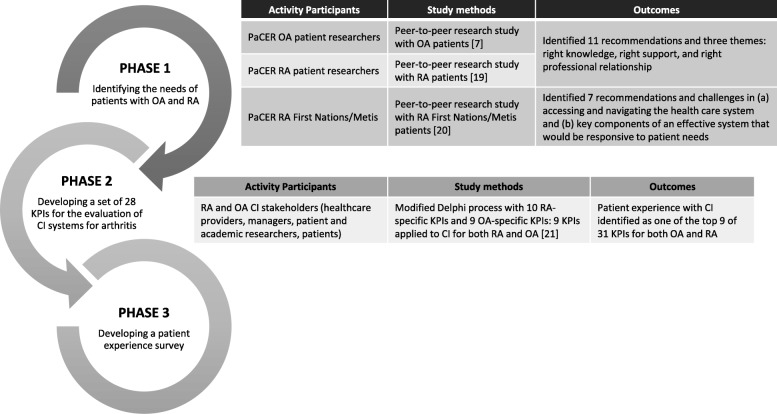
Summary of study outcomes from Phases 1 & 2

The starting point for this initiative was to identify the needs of patients with osteoarthritis, rheumatoid arthritis, and First Nations Metis and Inuit patients with rheumatoid arthritis (Phase1 – see Fig. 1). The Patient and Community Engagement Research (PaCER) group were asked to lead this phase of the research at the start of the research project [17]. The PaCER method is a peer-to-peer inductive research approach designed to create a robust collective patient voice and maximize patient engagement throughout the research process [7, 12, 13, 18]. Using the same process, patients with RA provided their perspectives on the challenges they face in accessing and navigating the health care system, and what they viewed as key components of an effective system that would be responsive to their needs [19]. The First Nations Metis and Inuit patients with rheumatoid arthritis provided several recommendations including the importance of clinic staff who understand their needs; the importance of developing wellness strategies specifically for this cohort of patients [20]. A total of 25 recommendations from the PaCER studies were identified.

These recommendations were then used to inform, in addition to other stakeholders, the development of a set of 28 Key Performance Indicators (KPIs) to evaluate centralized intake for arthritis care and the detailed process is reported elsewhere [21]. Stakeholder meetings including healthcare providers, managers, researchers, and patients with OA or RA provided input on potential KPIs and aligned with five of six quality dimensions from the Alberta Quality Health Matrix of appropriateness, accessibility, acceptability, efficiency, and effectiveness [22]. At this stage of the research cycle, patients and PaCER researchers were collaborating and contributing through participation and discussion to the research activity [17]. Literature reviews were conducted to ensure KPIs were based on best practices and harmonized with the six core performance measures of the National Arthritis Alliance of Canada’s Measurement Framework [23] and finalized using the RAND developed ExpertLens methodology [24]. The final phase involved a panel (*n* = 28) of patients, the patient engagement researchers from PaCER, care providers and system administrators, who prioritized KPIs using a Multi-Criteria Decision Analysis process [21]. Importantly, KPIs reflecting patients experience were ranked in the top 10 by patients, PaCER researchers and other stakeholders on the panel, with 95% rating it as 9/10 for importance [21]. (Phase 2 – see Fig. 1). These KPIs informed the development and evaluation of an optimal centralized intake system for arthritis care, as well as providing valuable indicators of quality of care for OA and RA patients. The findings also created the impetus to develop a patient experience survey as the patient experience was central to the evaluation of the central intake model (Phase 3).

Capturing the extent of patient involvement in the research process has uncovered some challenges, such as inadequate reporting of patient involvement, and a lack of valid and reliable tools to capture the impact of patient and public engagement [10]. The Guidance for Reporting Involvement of Patients and Public (GRIPP) checklist [25, 26] addresses this gap but has been criticized for assessing the impact of patient engagement in research, rather than the quality of the reporting [27]. Recognizing that there was limited understanding of how patients were being engaged in rheumatology research, a recent review sought to develop a framework to advance reporting of patient engagement [17]. They proposed a new framework based on a scoping review and analysis of 30 publications related to patient engagement. Their model, the Patient Engagement in Research Description (PED) framework, identifies three over-arching categories of who, how, and when, that should reported in relation to patient engagement in the research process. They use the term patient research partner (PRP) to describe patients, their family members, and informal caregivers who participate in health research initiatives. The ‘who’ should describe the affiliation and research-relevant health characteristics of the PRP. How they are engaged would describes the process of engagement and includes initiation of engagement, the method of contribution and level of engagement e.g. informed, consulted, collaborated and led. Finally, the category ‘when’ captures the different time points along the trajectory or life cycle of the research project. In describing the process of co-design in our study we also describe the who, how and when of patient engagement.

The specific aim of this paper is to describe our process of co-designing a patient experience survey for people with arthritis referred to a central intake clinic. For the future, the intention is to embed the patient experience survey in the routine evaluation of service redesign for centralized intake to improve arthritis care.

## Methods

### Study design

The design of the study is essentially one that reflects a constructivist approach where knowledge is constructed through collective approaches [28]. Engaging patients to capture their experiences in the design and re-design of healthcare services have been a central concept of improvement initiatives and includes core principles of equity, understanding experiences, and improving services [29]. In healthcare improvement co-design has been identified as an approach to participatory design, involving all stakeholders in a process to ensure the results meet their needs and is usable [30]. We have used the term co-design to describe the process of for developing the patient experience survey, collaboratively with patient researchers.

The process of developing the patient experience survey comprised of three connected phases: 1) Identifying the needs of patients with osteoarthritis and rheumatoid arthritis, 2) developing a set of key performance indicators for the evaluation of CI systems for arthritis, and 3) the co-design process for developing items for a patient experience survey for arthritis care. Phases 1 and 2 have been briefly described in the background as they were instrumental in the development of the survey and a summary of key outcomes from these two phases are provided in Fig. 1. For this study the patient experience survey was a core component of the baseline evaluation to be conducted in the future.

Institutional ethical review and approval were obtained for each of the phases (University of Calgary Conjoint Health Research Ethics Board ID: REB13–0822). In describing our process, we have attempted to make transparent the key elements of who, how and when, from the PED framework [17].

### Setting of the study

The study was carried out in an urban setting in Western Canada, within a single provincial healthcare organization. Physicians are paid on a fee-for-service (FFS) model. The central intake referral system for arthritis care in the province serves over 4.3 million residents.

### Participants

To identify items that would ultimately be included in the patient experience survey a small working group comprising of PaCER researchers (JM, ST) and academic researchers (*n* = 4) were convened from the broader research team. It was important to have patient stakeholders who could bring both their own experiences of living with arthritis but were also patient researchers and a part of the Patient and Community Engagement Research group [12]. The two PaCER researchers were co-investigators on the research team and had been involved in several studies in this program of research around the delivery of arthritis care. Both of them had previously conducted research with patients to ascertain what was important for people with OA seeking support for their symptoms [7]. The four academic researchers consisted of the principal investigator for the research who was a senior health services researcher and health economist. The two co-investigators responsible for the evaluation phase of the study had experience of mixed methods, qualitative research and health care evaluation, and the research coordinator was experienced in managing complex research studies. All the academic researchers had extensive experience of patient engagement in health research.

### Procedure and analysis

**Fig. 2:**
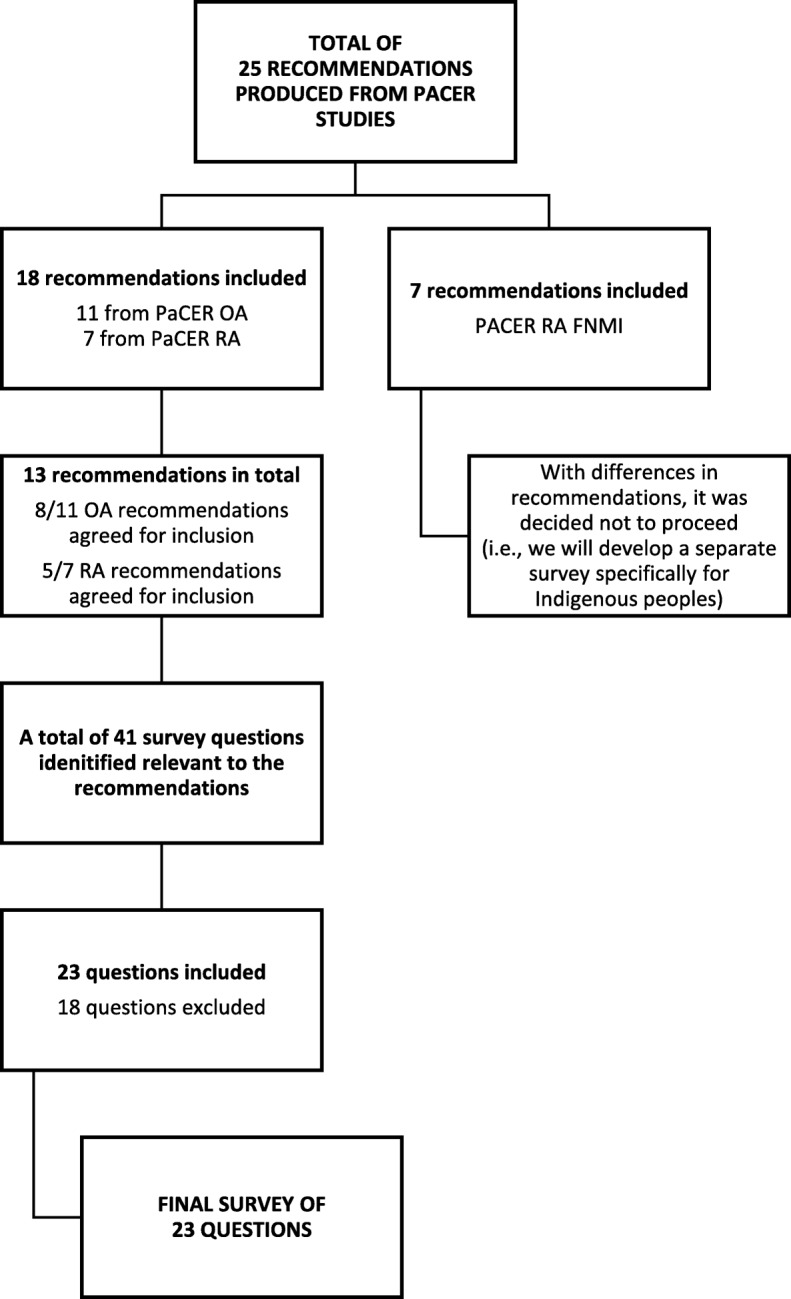
Decision making for item selection in the development of the patient experience survey

The working group participated in 10 bi-monthly one-hour telephone conference calls (Nov 2015 – April 2016) with follow up calls and email contact between meetings, as required. The purpose of these meetings was to develop the patient experience survey. To do this the meetings were very task focused with PaCER researchers contributing by participating all aspects of this process and completing research-related tasks i.e. rating survey items. The following two sections describe in detail the identification and selection of the patient survey items (see Fig. 2).

The first task was to identify which recommendations from the PaCER reports should be reflected in the patient experience survey. The working group were provided with the 25 recommendations from the three patient cohort reports i.e. OA (11 recommendations), RA (7 recommendations), and First Nations, Metis and Inuit RA cohort (7 recommendations) on a spreadsheet. They were then provided with definitions of each of the patient-identified themes created from the OA and RA PaCER recommendations (right knowledge, right professional support, and right professional relationships) [7, 19]. Following discussion with the working group an additional theme of ‘timeliness of care and communication’ was added to reflect the wider scope of the central intake process. The working group was asked to sort recommendations into one of the four themes. They were also asked to rate each recommendation as “yes”, “no”, or “maybe” for inclusion. These responses were first documented on their individual spreadsheet and sent to the research coordinator who collated the results onto a final group spreadsheet. The group then compared individual ratings, discussed items and came to a consensus for which ones to include/exclude.

The next stage was creating a pool of survey items from validated instruments as a starting point for generating a patient experience survey item pool. These included the Consumer Assessment of Healthcare Providers and Systems (CAHPS®) patient survey questions [31], the Patient Experience Survey instrument for the Canadian Institute for Health Information (CIHI) Primary Health Care Survey project [32], rheumatoid arthritis outpatient satisfaction survey [33], and the UK’s National Health Service Outpatient Questionnaire [34]. Forty-one survey items were identified as relevant to the 13 patient recommendations and screened to ensure none replicated PaCER recommendations and were not outside the scope of the project (JP/DM). This pool of items was then shared with the working group for a mapping exercise to determine which items would be included and identify gaps. Again, a rating of ‘yes’, ‘no’ and ‘maybe’ was used. Similarly, the task was completed individually, and then as a group, by comparing ratings and discussing items. Similar to the process for identifying which recommendations should be reflected in the patient experience survey, the responses were first documented on their individual spreadsheet and sent to the research coordinator who collated the results onto a final group spreadsheet. The group then compared individual ratings, discussed items and came to a consensus for which ones to include/exclude.

Finally, the updated list of questions along with a standardized scoring method was compiled into a draft survey and presented to arthritis patients at one clinic (*n* = 4) who provided feedback on the contents of the survey. They were asked to comment on the clarity of questions, length of the questionnaire, and suggestions for change.

## Results

The first task of identifying which of the 25 recommendations from the PaCER reports should be included in the survey resulted in 3 OA and 2 RA recommendations being excluded as being out of scope e.g. recommendation for the development of clinical guidelines, and recommendations for further research. There was minimal discrepancy between the raters in their evaluations with the exception of the 7 recommendations from the First Nations, Metis and Inuit (FNMI) cohort. Whilst the patient-researchers recommended inclusion, the academic researchers had excluded them, as several of these were particularly unique to the cohort and not common for both RA and OA cohorts. For example, incorporating traditional wellness strategies was particularly important [35] and yet the need for peer support, identified by other cohorts as important, was not highlighted as a need [20]. Given the challenges to include recommendations that were not common across the other two cohorts it was decided that a separate patient experience survey for this group capturing specific requirements, such as the delivery of services based on indigenous approaches to health and healing, will be developed at a future time. It was also important to ensure that survey items would reflect the recommendations from the Health Quality Council of Alberta, for improved patient experience and outcomes: accurate information between providers and patients; maintaining continuity between providers; understanding the individual responsibilities of providers; and ensuring patients are aware of who to contact for assistance [36]. A total of 13 recommendations were made for quality arthritis care, from the PaCER reports for patients with osteoarthritis and rheumatoid arthritis.

From the pool of survey items from validated instruments the rating process eliminated a total of 18 items from the patient experience survey item pool for the following reasons: replication of PaCER recommendations (5), outside the scope of the project (10), included activities patients would not experience e.g. laboratory results (3). Feedback from the four arthritis patients at one clinic led to minimal changes and the survey was considered complete (see Additional file 1). The final patient experience questionnaire had a total of 23 items.

## Discussion

Engaging patients or users, to capture their experiences in the design and re-design of healthcare services has been a central concept of improvement initiatives and includes core principles of equity, understanding experiences, and improving services [29]. In healthcare improvement co-design has been identified as an approach to participatory design, involving all stakeholders in a process to ensure the results meet their needs and is usable [30]. A variety of activities have been identified as part of this process, such as gathering experiences, bringing patients and healthcare staff together, and using triggers to identify shared priorities for improvement. In this paper, we use the term ‘co-design’ to describe our process of patient engagement to co-design a patient experience survey for people with arthritis referred to a central intake clinic. From this process, we identify several aspects that bring a unique perspective and warrant discussion. Firstly, the process of patient engagement in co-designing a survey identified indicators of arthritis care that had not featured in validated surveys related to the patient experience of health care. Secondly, the concept of integrated care appears to be a core need from the patients’ perspective in terms of the delivery of high quality arthritis care. Thirdly, the different roles in co-design and some of the issues that warrant further consideration. Finally, suggestions for future directions and some of the limitations and challenges of this work are explored.

In this discussion, we draw on the framework offered by Hamilton and colleagues to describe who the patients were, how we engaged with them and at what stages of the research cycle [17]. The model of patient engagement was primarily from Patient and Community Engagement Research (PaCER), as well as patients outside this group, and led the initial phase to identify what elements of arthritis care were important to them, using an inductive research approach [7, 19]. Findings from these initial studies were used to inform, along with other stakeholders, the development of a set of 28 Key Performance Indicators (KPIs) to evaluate centralized intake [21]. The importance of the patient experience, identified by all stakeholders as one of the top 10 KPI’s, led to the development of the survey. Hamilton’s model [17] was helpful in providing descriptors to identify important components for reporting patient engagement but the linear organization often belies the complexity of reality. In particular, we found a need for flexibility in the level of engagement with patient researchers. Patient researchers moved from collaborating, then leading, and then collaborating, depending on the task. Recognising the importance of flexibility in the model and the interplay between descriptors, around how engagement takes place is important. Our experience suggests that involving the end user is essential for integrated knowledge translation and capturing meaningful data through participation with others, reflection, and a goal of pursuing practical solutions for issues that concern people [37]. These initiatives intend to close the gap between research and practice. In the United States, recognizing the significant gap between research and application to clinical practice, a new taxonomy for patient engagement in patient-centered outcome research sought to reduce this discrepancy [36]. However, prior to external review with key stakeholders, the co-authors created three drafts with no patient stakeholder involvement, although they did in further iterations. Unless the patient stakeholder is involved at the outset it may be difficult to shape the future content or direction of outputs. In our study, the level of engagement for this stage was collaborative and iterative where decision-making was shared between patient and academic researchers [17]. We experienced minimal discrepancy between individual ratings for both the PaCER recommendations and survey item mapping, with the exception of those recommendations from the First Nations, Metis and Inuit cohort. This discrepancy perhaps reflected the academic researchers being more cognizant of the limited research resources available. However, during this process, we were surprised to find that several PaCER recommendations were not covered in the validated instruments survey items. This necessitated the development of survey items from these recommendations (see Table 1 for examples). This also resulted in a shift in the level of engagement, whereby the patient researchers were assigned authority over decision-making for an aspect of the research process, but executive power remained with other stakeholders [17]. Our initial premise being that these validated instruments would have captured all the important items. Yet closer scrutiny of one validated survey - Consumer Assessment of Healthcare Providers and Systems (CAHPS®) [31] - state that ‘patients with chronic conditions were represented’ in each of the four focus groups used to create an item pool. However, no details of the chronic conditions, and whether any of these patients had arthritis, were provided. This gap underlines the importance of ensuring that end users are involved at the earliest opportunity in the development of a survey to facilitate the generation of high quality data.

**Table 1:** Recommendations for high quality healthcare for OA patients from PaCER report^a^ and example survey items

Recommendations	Example survey items
Patient needs right knowledge: • Has to be comprehensive, detailed, and no-nonsense. • Knowledge on disease progression, and corresponding evidence-based management strategies • Information on when should we seek help and from whom • What can the patient do when something no longer works.	• The care providers at the clinic explained the proposed treatment plan to me in a way I could understand • The care providers at the clinic responded to all my questions or concerns in a way I could understand • The providers at the clinic explained to me what to do if my arthritis gets worse
Patient needs right professional support: • Access to publicly funded evidence-based resources • Toolbox of disease management strategies at primary care level • Access to OA and RA expertise	• I received information on other options to manage my arthritis (e.g. physiotherapy, acupuncture, chiropractor, non-medical wellness strategies) • It was difficult to reach the care providers at the clinic
Right relationship between patients and care providers: • Able to come back to someone who knows patients to assist to effectively self-manage • Remember patients as individuals • Remember patient’s previous visits • Personalized and evolving self-management plan embedding patient choices	• The care providers at the rheumatology clinic respected my wishes and ideas about my treatment • The care providers made efforts to understand what having arthritis means to me • My care was well-coordinated among different care providers at the rheumatology clinic

^a^Recommendations from the report for each of the three themes (Miller et al. 2016) [7]

The patient experience survey centered on three themes derived from arthritis patient’s needs: the right knowledge, the right professional support and the right professional relationship [7]. Together these themes reflect the concept of integrated care, that has been defined as “patient care that is coordinated across professionals, facilities, and support systems; continuous over time and between visits; tailored to the patients’ needs and preferences; and based on shared responsibility between patient and caregivers for optimizing health” [38]. Patients clearly understand the important components for integrated care and identify when integration and coordination of care, do or do not happen, in their experiences with the healthcare system [39]. The importance of integrated or seamless care across primary and secondary centers for arthritis care, along with other long-term conditions has been highlighted [40, 41] and adopted widely, including Europe [42, 43], Australia [44], New Zealand and Canada [45]. A systemic review of integrated primary-secondary care identified improved elements of disease control and service delivery at a modestly increased cost, although the impact on clinical outcomes was reported to be limited [46]. Interestingly, reduced health care costs were a prominent feature in a systematic review of integrated care models for patients with chronic disease [47]. It is curious to ask what might be the outcomes that patients would value being measured, beyond clinical and cost? Unfortunately, barriers to integrative care models are extensive, often requiring organizational changes, greater personal and professional collaboration and primary care physician education [40]. In Alberta, Canada, a facilitated model of integrated care to improve arthritis detection and treatment, in an urban Aboriginal population, was successfully embedded in a primary care setting [48]. The Alberta Health Services 2017–2020 business plan has prioritized improving the experience of patients and families, and for the first time includes the specific goal of ‘ensuring our health system is integrated and coordinated between providers and patients’ [49]. Details on how this will be achieved are limited but it provides a strong impetus for the co-design of new systems for integrated care.

The process of co-design requires careful consideration of several factors, including when to involve patients in the research process, how to involve them and some of the potential challenges involved. For this study the two patient researchers had been involved from the outset, both in the earlier OA and RA PaCER studies (phase 1), and the development of the KPIs (phase 2). Engaging patients in all phases of the research study is seen as highly desirable [50]. Pragmatically, the process incurred challenges that have been well reported in the literature relating to patient engagement. Meaningful patient engagement incurs higher use of resources around time, costs and support [51, 52] and brings additional logistical challenges [50]. However, we believe the benefits outweigh the constraints around complexity and additional timing that co-design requires. Constructing a team with expertise in meaningful patient engagement, methodology and including patient researchers is essential for the production of high quality data.

### Future directions

Patient engagement and measuring the patient experience is recognized as fundamental to enhancing service delivery, but care providers must also be engaged to listen and take action on patient feedback. The development of high quality patient experience data needs to be matched by the receivers (healthcare providers) being receptive to the information, to then close the loop and make the co-design of healthcare a reality. The role and importance of effective communication in quality improvement has been emphasized [53, 54], but systematic and structured communications strategies are not usually formally integrated into quality improvement initiatives [55]. As we become more sophisticated at developing high quality patient experience surveys, we suggest that new approaches are required close the feedback loop, by engaging health providers and creating the impetus for quality improvement. We have described our process of patient engagement to co-design a patient experience survey for people with arthritis referred to a central intake clinic. Using the Patient Engagement in Research Description (PED) framework to describe in more detail the process of patient engagement. This continues to be a work in process that will feed into our larger project and future work and firmly situates the patient and their experiences at the center.

### Limitations

There were several limitations to the study. The survey requires further development and testing, beyond establishing face validity, to ensure it is a validated and robust tool for measuring the patient experience of central intake [56, 57]. In terms of developing items for the patient experience, we were less successful in identifying those related to patients needs around professional support. This warrants further exploration, from a patient perspective, to understand what sort of professional support could be offered in primary care, prior to referral to CI. The operationalization of the patient experience survey for this population is currently being explored and will contribute to the validity of the survey.

## Conclusion

The purpose of this paper was to describe the process of patient engagement to co-design a patient experience survey for people with arthritis referred to central intake. Patient engagement and co-design of a patient experience survey identified aspects of care that had not been previously recognized. The concept of integrated care is a core requirement from the patients’ perspective for the delivery of high quality arthritis care. Frameworks to describe the level of patient engagement in research are helpful for reporting this activity but need to recognize the non-linearity of the process. New approaches are needed to prepare and facilitate care providers to receive and act upon data from patient experience surveys. Additional resources of cost, time and expertise for patient engagement in co-design activities are recognized and should be included, where possible, to ensure high quality data are captured.

## Additional file

Additional file 1: Patient Experience Survey. (DOCX 37 kb)

## Data Availability

The datasets generated and/or analysed during the current study are not publicly available due to the complexity of the multi-phased study but are available from the corresponding author on reasonable request.

## Abbreviations

AHS: Alberta Health Services
AI: Alberta Innovates
CAHPS: Consumer Assessment of Healthcare Providers and Systems
CI: Central Intake
FNMI: First Nations, Metis and Inuit
GRIPP: Guidance for Reporting Involvement of Patients and Public
KPIs: Key Performance Indicators
OA: Osteoarthritis
PaCER: Patient and Community Engagement Researchers
PED: Patient Engagement in Research Description
PRP: Patient Research Partner
RA: Rheumatoid arthritis
YLDs: Years Lost to Disability
