# Supplementary material for: Co-design of a patient experience survey for arthritis central intake: an example of meaningful patient engagement in healthcare design
Source: BMC Health Serv Res. 2019 Jun 4;19:355. doi: 10.1186/s12913-019-4196-9 (PMC6549374; doi:10.1186/s12913-019-4196-9)
Supplement: Supplementary file 1 — Patient Experience Survey. (DOCX 37 kb) [file 12913_2019_4196_MOESM1_ESM.docx]

Additional file 1

## Patient Experience Survey

| Statement | Strongly Agree | Agree | Disagree | Strongly Disagree | Not  Applicable |
| --- | --- | --- | --- | --- | --- |
| 1. Care for my arthritis started quickly after the referral to the clinic | □ | □ | □ | □ | □ |
| 2. The referral from my family doctor to the clinic was dealt with in a timely manner | □ | □ | □ | □ | □ |
| 3. It was difficult to reach the care providers at the clinic | □ | □ | □ | □ | □ |
| 4. The care providers at the clinic knew important information about my medical history | □ | □ | □ | □ | □ |
| 5. My family doctor is informed and up-to-date about the care I receive at the clinic | □ | □ | □ | □ | □ |
| 6. My care was well-coordinated among different care providers at the clinic | □ | □ | □ | □ | □ |
| 7. I received consistent messages from all of the different care providers at the clinic | □ | □ | □ | □ | □ |
| 8. The care providers at the clinic respected my wishes and ideas about my treatment | □ | □ | □ | □ | □ |
| 9. I was as involved as I wanted to be in making decisions about my treatment for arthritis | □ | □ | □ | □ | □ |
| 10. The care providers at the clinic asked me about my goals for treatment and what is important to me in managing my arthritis | □ | □ | □ | □ | □ |
| 11. The care providers at the clinic responded to all my questions or concerns in a way I could understand | □ | □ | □ | □ | □ |
| 12. The care providers at the clinic explained the proposed treatment plan to me in a way I could understand | □ | □ | □ | □ | □ |
| 13. Before my treatment for arthritis, all the risks and/or benefits were explained to me in a way I could understand | □ | □ | □ | □ | □ |
| 14. The care providers at the clinic explained the reasons for all the tests in a way I could understand | □ | □ | □ | □ | □ |
| 15. The care providers at the clinic explained my test results to me in a way I could understand | □ | □ | □ | □ | □ |
| 16. The purpose of the medications that were prescribed for arthritis were explained to me in a way I could understand | □ | □ | □ | □ | □ |
| 17. The information I received about arthritis was clear | □ | □ | □ | □ | □ |
| 18. I received information on other options to manage my arthritis (e.g. physiotherapy, acupuncture, chiropractor, non-medical wellness strategies) | □ | □ | □ | □ | □ |
| 19. The care providers at the clinic gave me information on how to self-manage my arthritis | □ | □ | □ | □ | □ |
| 20. The care providers at the clinic explained to me what to do if my arthritis gets worse | □ | □ | □ | □ | □ |
| 21. The information I received on peer support groups for arthritis was useful | □ | □ | □ | □ | □ |
| 22. Overall, I was treated with respect while I was at the clinic | □ | □ | □ | □ | □ |
| 23. The care providers at the arthritis clinic made efforts to understand what having arthritis means to me | □ | □ | □ | □ | □ |

**Additional comments:**

­­­­­­
